# The Effect of Polarity and Hydrostatic Pressure on Operational Characteristics of Rutile Electrode in Underwater Welding

**DOI:** 10.3390/ma13215001

**Published:** 2020-11-06

**Authors:** Andrés M. Moreno-Uribe, Alexandre Q. Bracarense, Ezequiel C. P. Pessoa

**Affiliations:** 1 Programa de Pós-Graduação em Engenharia Mecânica—PPGMEC, Robotics, Welding and Simulation Laboratory—LRSS., Federal University of Minas Gerais—UFMG, Belo Horizonte 31270-901, Brazil; queiroz@demec.ufmg.br; 2 Welding/Materials Joining Engineering Department, LeTourneau University, 2100 S. Mobberly Avenue, Longview, TX 75602, USA; EzequielPessoa@letu.edu

**Keywords:** underwater welding, wet welding, polarity, arc phenomena, weld bead morphology, SMAW

## Abstract

In order to provide a better understanding of the phenomena that define the weld bead penetration and melting rate of consumables in underwater welding, welds were developed with a rutile electrode in air welding conditions and at the simulated depths of 5 and 10 m with the use of a hyperbaric chamber and a gravity feeding system. In this way, voltage and current signals were acquired. Data processing involved the welding voltage, determination of the sum of the anodic and cathodic drops, calculation of the short-circuit factor, and determination of the melting rate. Cross-sectional samples were also taken from the weld bead to assess bead geometry. As a result, the collected data show that the generation of energy in the arc–electrode connection in direct polarity (direct current electrode negative-DCEN) is affected by the hydrostatic pressure, causing a loss of fusion efficiency, a drop of operating voltage, decreased arc length, and increased number of short-circuit events. The combination of these characteristics kept the weld bead geometry unchanged, compared to dry weld conditions. With the positive electrode (direct current electrode positive-DCEP), radial losses were derived from greater arc lengths resulting from increasing hydrostatic pressure, which led to a decrease in weld penetration.

## 1. Introduction and Theoretical Approach

The stick shielded metal arc welding (SMAW) process applied to aquatic environments is used as a low-cost practical repair and maintenance solution. Rowe and Liu (2001) [1] stipulated that the primary industries where it is applied are oil and gas, with the repair of offshore structures and the installation of pipes being the main projects involved.

However, the characteristics of underwater environments generate defects that affect the quality of the resulting weld joints and, thus, the mechanical properties of the structures. Therefore, essential variables must be taken into account, as they have a direct influence on the wet weld. Some inherent aspects of the process that need to be considered include the instability of the electric arc resulting from increased hydrostatic pressure and other environmental factors [2]; further, Ando and Asahina (1983) [3] mentioned that water dissociation promotes the absorption of hydrogen and oxygen by the weld metal, Pope et al. (1996) [4] noted the exposure of the weld metal and heat affected zone (HAZ) to rapid cooling due to the aqueous medium, and Suga and Hasui (1990) [5] referred to the decrease in the electrode fusion rate with increasing pressure. Also, the choice of waterproof coating results in lower diffusible hydrogen content in deposited metal, as mentioned by Tomków et al. (2020) [6]. Recently, interest in the effect of water depth on the diffusible hydrogen content has increased significantly, as shown by Klett et al. (2020) [7] and Chen et al. (2020) [8] Thus, the intricacies of this technique and knowledge of its main limitations have been studied by many researchers.

In this context, many studies have been developed to describe the physical and operational aspects of underwater wet welding (UWW) with stick electrodes. Dupont et al. (2013) [9] stated that details of the fusion pool chemistry of SMAW processes have been characterized and are well understood; however, some aspects have not been clarified (e.g., understanding the polarity effect on the weld bead morphology and the melting rate of the consumable in wet conditions). Pessoa (2007) [10], who characterized the geometry of bead-on-plate welds made with electrodes E6013 and E7018 at 50 and 100 m water depth, reported a higher weld penetration and lower electrode melting rate for the DCEN setting. This particularity was also described by Szelagowski et al. (1992) [11] and Grubss et al. (1996) [12]; however, the reversal of the arc characteristics in the aqueous medium, which results in less penetration in DCEP (compared to DCEN) with the use of coated electrodes, has not yet been explained, according to Mazaferro (1998) [13].

Tsai and Masubuchi (1977) [14] presented a model for UWW which is related to the change in arc characteristics with the polarity of the process and, consequently, with the possible changes in the shape of the weld bead. For underwater welding with a coated electrode, they concluded that polarity is the controlling factor in penetration. In this way, it was shown that direct polarity provides narrow weld beads with deep penetration as a result of the heat generated in the arc, which is directed to the melt pool and acts on the base metal. On the other hand, in reverse polarity, the heat generated by the arc is directed to the electrode, such that its core rod melts more quickly, causing a higher deposition rate and less penetration. These descriptions were based mainly on the structure of the electric arc and its heat distribution proposed by Tsai and Masubuchi (1977) [14] where, for the DCEN configuration, 80% of the heat goes to the anode, 10% is removed by the gases generated by the melting flow of the coating, and the remaining 10% goes to the cathode.

Considering the previous model from the thermal point of view, it is possible to find similarity with the energy balance in the Gas Tungsten Arc Welding (GTAW) process using DCEN in air welding where, according to Giedt et al. (1989) [15], approximately 60–80% of the heat generated is absorbed into the anode, generating greater fusion volume, compared with the reverse polarity situation. However, unlike GTAW, in which the generation of electrons occurs by thermionic emission (in DCEN), Essers and Walter (1981) [16] reported that, when welding with consumable electrodes, electrons are emitted by field emission phenomena (in both DCEN and DCEP). Thus, in conventional electric arc welding with a consumable electrode (SMAW; Gas Metal Arc Welding, GMAW; Submerged Arc Welding, SAW), the electrode connected to the negative pole incurs significant differences in relation to its use in DCEP configuration (Cirino and Dutra, 2010) [17]. These differences are most notable in the melting rate, metal transfer mode, and the resulting weld bead geometry. Thus, for example, Waszink and Piena (1985) [18], considering the thermal processes in coated electrodes applied in air welding, stipulated that heat generation is dominated by the cathode and anode phenomena that occur at the tip of the electrode and next to the base metal.

According to Allum (1982) [19], there are essentially two components that influence the heat in the anode: electronic heating related to the flow of electrons into the anode and gas heating. Equation (1) shows the anode heating (*Qa*) due to electron condensation:

*Qa* = *[(3kT/2e)* + *ϕ* + *Ua]I* (1)

where *3kT/2e* is the electron thermal energy in the high-temperature plasma, *ϕ* is the anode thermionic work function, and *Ua* is the anode fall space voltage.

The phenomena that occur at the cathode, although similar, have a different effect. For these, the heat generated (*Qc*) is estimated using Equation (2), where the negative signs represent losses. Fuerschbach (1998) [20] refers to this heat loss as a cooling effect; at the cathode, part of the heat generated, *UcI*, is spent to emit *ϕI* and accelerate the electrons (by *3kT/2e*).

*Qc* = *[(−3kT/2e)* − *ϕ* + *Uc]I* (2)

Scotti et al. (2012) [21] established that the heat produced in the cathodic zone represents most of the heat in the arc; consequently, if the electrode is supposed to be connected to the positive of the welding machine (DCEP), the cathodic drop region is close to the workpiece and the amount of energy diffused by the workpiece (*Qc*) results in greater heating and fusion. Thus, Wainer et al. (1992) [22] concluded that greater penetration should be expected with this polarity configuration.

However, with changes in the cathode and anode location, less penetration and higher electrode fusion rate would occur in DCEN, as defined by Quites and Dutra (1979) [23].

For Marques and Modenesi (2014) [24], the rate at which the electrode is melted is important in assessing the productivity of a process. Lesnewich (1958) [25] developed an empirical equation that is valid for operating conditions where the welding current is kept approximately stable, and which makes it possible to analyze the variables that affect the electrode melting rate (Equation (3)):

*w* = *α I* + *βsI^2^* (3)

where *w* is the melting rate (mm/s), *I* is the welding current (A), and *s* is the length of the electrode (mm). The term *βsI^2^* represents the contribution of the joule effect to the fusion of the electrode-wire, while the term αI represents the contribution of heating of the arc fall region (anode or cathode, depending on the polarity setting).

Experimental results obtained by Lesnewich (1958) [25] in GMAW have indicated that the *α* coefficient for positive electrode welding is, in a first approximation, independent of the welding current, composition of the shielding gas, arc length, welding voltage (Nunes, 1982) [26], the surface conditions of the wire, and the pressure.

In GMAW welding with the negative electrode, Lancaster (1984) [27] mentioned that the *α* coefficient tends to be higher than when welding with the positive electrode, where this difference can be up to 75%. Thus, Scotti and Ponomarev (2008) [28] defined that the fusion rate in DCEN can be up to 70% higher than that in relation to the electrode configuration on the positive pole for GMAW welding.

However, and unlike the DCEP polarity configuration, when the electrode is connected to the negative pole, Modenesi (2015) [29] mentioned that *α* can be reduced to values close to or even lower than those with positive electrode welding by using special coatings on the wires and when conducting hyperbaric welding.

According to the previous considerations, which support the characteristics of electric arc welding processes with consumable electrodes, the objective of this research is to propose a model that explains the weld bead geometry and the consumable fusion rate as a function of the polarity configuration. For this, we used a rutile-based electrode which, according to Arias and Bracarense (2017) [30], is a consumable that is widely used in UWW and which presents, in a remarkable way, the effects of depth on its performance, according to Brown et al. (1974) [31].

## 2. Materials and Methods

### 2.1. Equipment and Materials

**Figure 1:**
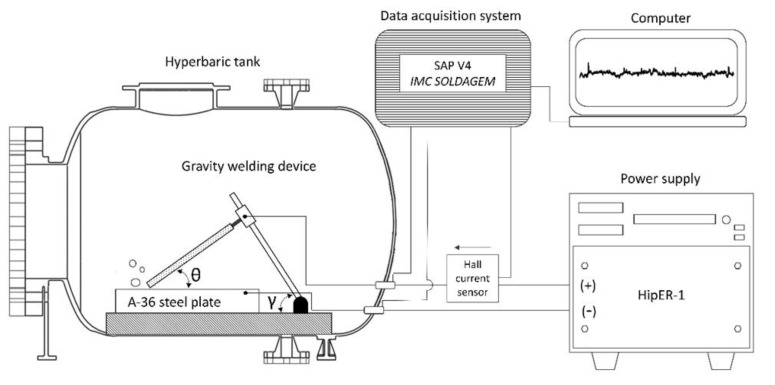
Diagram of the welding jig. Uribe et al. (2017) [32] defined that the angle θ is related to the welding speed and the angle γ is related to the component of gravitational force acting on the electrode fixation claw. Adapted from the work in [32].

Bead-on-plate welds were performed with the use of a gravity welding system and inside of a hyperbaric welding simulation chamber that could be pressurized up to 20 kgf/cm^2^ (equivalent to approximately 200 m underwater). During all tests, the welds were carried out using the “drag technique” in tap water and the configuration angles of the mechanized welding system were θ = 60° and γ = 80° (Figure 1). The electrode angle and the bar angle used in this study were the settings that showed the best bead morphology and continuity. A constant current power source for subsea operations was used, and voltage and current welding signals were acquired at a rate of 5 kHz.

In this study, 6.35 mm thick ASTM A-36 steel plates were used as specimens in the tests. A rutile electrode with a nominal diameter of 3.25 mm and a length of 350 mm was used for underwater welding. The consumable was waterproofed with two layers of vinyl varnish.

### 2.2. Methodology

#### 2.2.1. Welding Test

Welds were made in the air (air welding, inside the hyperbaric chamber) and at the simulated depths of 5 m (0.5 kgf/cm^2^) and 10 m (1 kgf/cm^2^) using reverse polarity (DCEP) and direct polarity (DCEN). For each combination of parameters (air welding or wet welding and polarity), six welds were made. The welding current used for both polarities and each of the conditions (air welding and wet welding) was 170 A. This value was previously determined through laboratory tests and corresponded to the best parameters found in the consumable performance study performed at the same depths by Uribe et al. (2017) [32].

In order to evaluate the morphology of the resulting welds, 10 mm thick traverse samples were taken from the center of the weld bead. These samples were mounted and prepared (according to conventional metallographic preparation techniques) to reveal the penetration, width, and reinforcement of the weld bead. Then, using commercial software (ImageJ, ImageJ bundled with 64-bit Java 1.8.0_172, National Institutes of Health-NIH, Bethesda, MD, USA), the main geometric characteristics of the weld bead were measured. Finally, the electrode melting rate was calculated following the methodology applied by Pessoa et al. (2010) [33]: measuring the length of the electrode consumed during the test and dividing it by the welding time.

#### 2.2.2. Data Processing

In order to evaluate the electrical signals resulting from the welding process, the data files were treated using the Sinal software (Developed by PhD. Paulo J. Modenesi). For this processing, the interval between the tenth and fifteenth seconds of the total welding time was considered, in order to neglect disturbances resulting from the opening of arc, thus ensuring a range of readings where the welding process was under an approximately stationary regime. Data processing involved measuring average values of the welding voltage (Ū), determining the sum of anode and cathode falls (Ua+c), and calculation of the short-circuit factor (*Fcc*).

**Figure 2:**
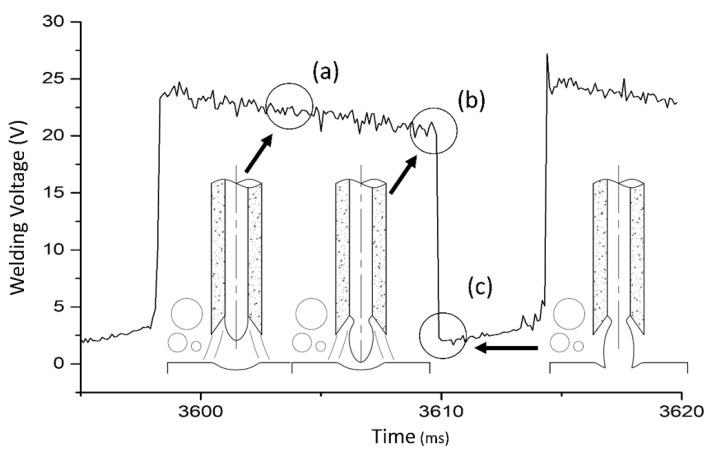
Methodology to determine Ua+c (adapted from the work in [5]): (a) welding voltage while the arc is operating, (b) instant immediately before the short circuit, and (c) moment immediately after the short circuit. At point (b), the arc length is close to zero, the voltage drop in the arc column can be neglected, and the difference between the voltages at the indicated points provides an estimate of Ua + c [35].

Values of the sum of anode and cathode falls (Ua + c) were estimated from voltage oscillograms of welding tests with metal transfer by short circuits. For this determination, the difference between the voltage at point (b)—immediately before the start of a short circuit—and that at point (c)—immediately after the short circuit was established—was used (see Figure 2). Further details of this methodology can be found in the work published by Modenesi et al. (2011) [34].

**Figure 3:**
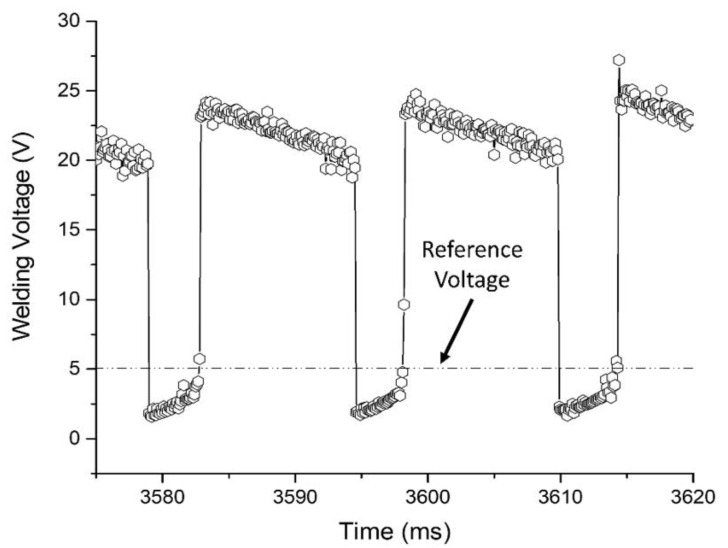
Schematic representation of the definition of the metal transfer period (*T*), short-circuit time (*tcc*), and reference voltage. Adapted from the work in [36].

The short-circuit factor (*Fcc*) was calculated using Equation (4). For this, the reference voltage of 5 V was defined based on the recommendation of Mazzaferro (1998) [13], and the Sinal software was used to evaluate the mean period of metal transfer (*T*) and the mean duration of the short-circuit period (*tcc*) from the voltage data, as shown in Figure 3. A condition of rejection of short-circuits of minimum duration (a single point) was also imposed, in order to minimize the influence of small voltage fluctuations in the results (e.g., those derived from fast short circuits).

*Fcc* = *(tcc⁄T) 100 (%)* (4)

#### 2.2.3. Electrode Tip Analysis

**Figure 4:**
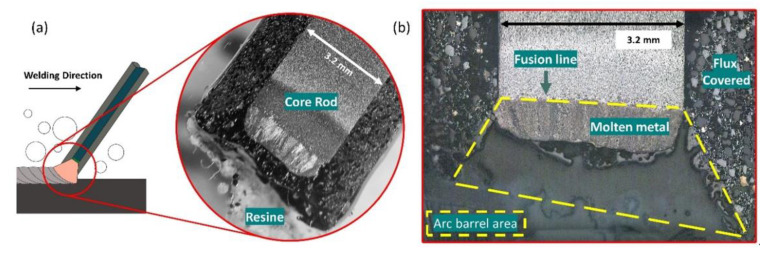
Scheme of the methodology for the measurement of arc barrel area in the tip of an electrode: (**a**) Arrangement of underwater wet welding process and a macrograph of the tip of an electrode; (**b**) image of the tip of an electrode showing the arc barrel area.

To estimate the size of the electric arc, measurements were taken of the arc barrel area at the tip of the coated electrodes (Figure 4). The electrodes were collected after 15 s of welding, at which point the electric arc was extinguished. The tips of the electrodes (with lengths of approximately 10 mm) were cut and mounted in resin. These samples were the sanded in the longitudinal section until reaching the nominal diameter of the consumable (3.2 mm), then polished and etched with Nital 2%. Micrographs were performed using an optical microscope (Leitz–Metallux II, Wetzlar, Germany). The area of the electric arc was measured from the melting line (division between molten metal and electrode tip ZTA) to the edge of the arc barrel using the ImageJ software.

## 3. Results and Discussion

### 3.1. Polarity and Pressure Effect on Arc Phenomena

#### 3.1.1. Voltage Welding and Characteristic Arc Length

**Figure 5:**
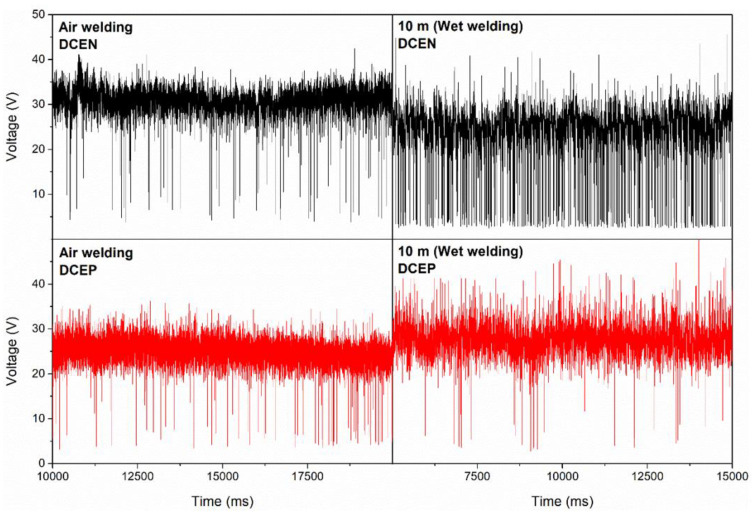
Welding voltage oscillograms for tests carried out in the air and at a depth of 10 m with a rutile electrode and for both polarities.

Figure 5 shows typical oscillograms for air welding and at a depth of 10 m with both polarities. It is possible to observe that the presence and intensity of short circuits varied, mainly with polarity and depth. It appears that an increase in depth increased the number of short circuits for direct polarity; meanwhile, for the reverse polarity, a possible increase in the arc length occurred and a greater amplitude of voltage fluctuation was observed.

**Figure 6:**
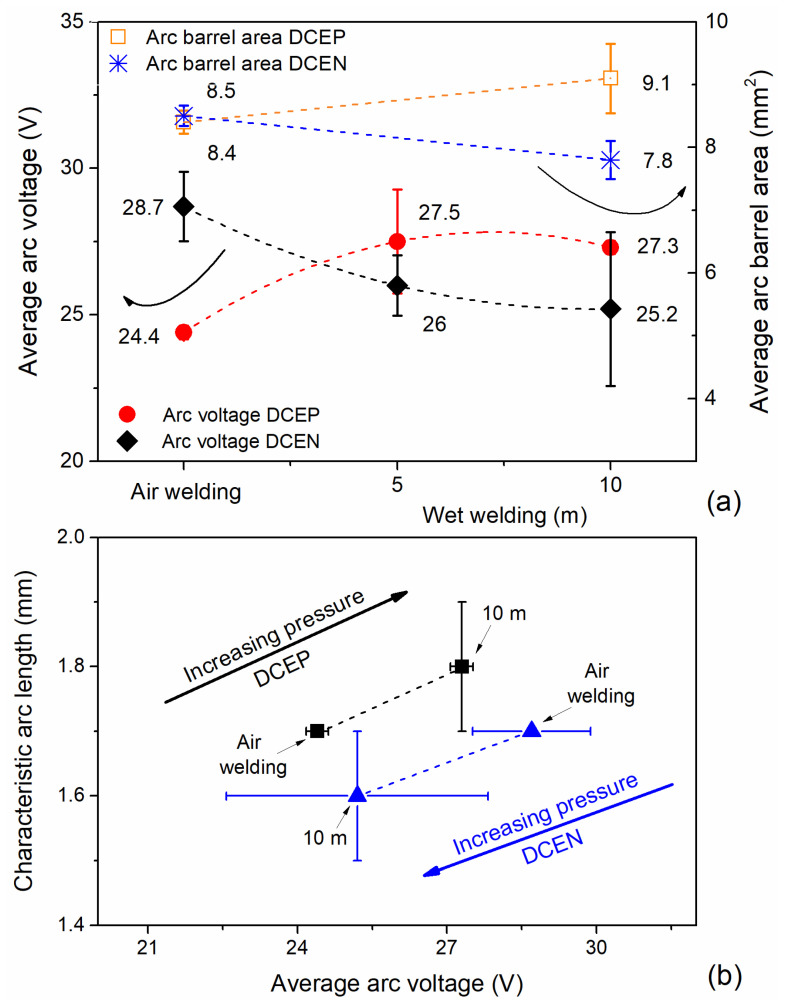
(**a**) Average arc voltage and arc barrel area for the rutile electrode in both polarities and for all conditions; (**b**) characteristic arc length vs. average arc voltage. Error bars show the standard deviation.

Aiming to not reach a hasty conclusion and in order to elucidate the electric arc phenomena, Figure 6a shows the average welding voltage as a function of polarity and environmental conditions. For air welding, DCEN provided higher operating voltages compared to reverse polarity. Furthermore, with increasing operating depth, there was a drop in the value of the average welding voltage in the negative polarity, reaching its lowest point at 10 m. Omajene et al. (2014) [37] reported that the welding voltage decreases with depth, particularly for DCEN, and that this behavior seems to be associated with the variation in the occurrence of short circuits during the process.

In the DCEP configuration, the operating voltage increased with depth; Liberato et al. (2018) [38] mentioned a similar behavior. The authors described that, for E6013 with the positive electrode, the amount of short-circuits dropped in wet conditions and the operating voltage increased by 30%, compared to shallow water conditions.

**Figure 7:**
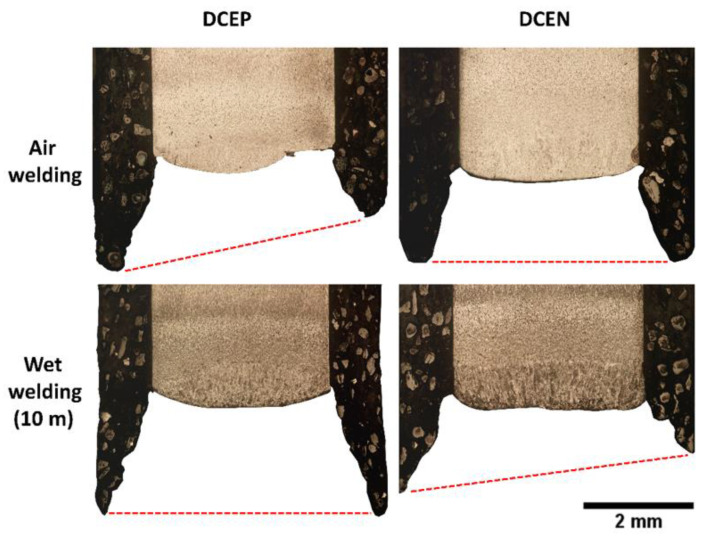
Images of the tips of electrodes showing the arc barrel.

According to Tsai and Masubuchi (1977) [14], for wet condition DCEP, the cone formed at the tip of the electrode was larger, which represents higher operating voltages and arc lengths. Thus, assuming that the voltage in the column is proportional to the arc length, Figure 7 shows images of the longitudinal section of the electrode tip for both polarity configurations. From the figure, it is possible to observe the shape of the cone trunk—representing the area of the electric arc at a certain point in the welding process—for the four tested conditions.

Figure 6a allows us to correlate the area of the electric arc with the average welding voltage already mentioned. In air welding, the area of the arc barrel did not change with the polarity; however, at 10 m depth, there was a clear trend in decreasing the area of the electric arc in DCEN, demonstrating the voltage drop with increasing depth. In DCEP polarity, the area of the electric arc rose with increasing depth.

From the area of the electric arc, it was possible to obtain the characteristic arc length, which was determined using the ratio between the arc barrel area and the diameter of the coating (5 mm ± 0.05). Figure 6b lists the arc length as a function of the average arc voltage. It is possible to visualize the direct relationship between the operating voltage and the arc length as a function of the simulated depth. Both the characteristic arc length and the average welding voltage clearly demonstrated the effects of pressure and polarity on the electric arc phenomenon in underwater welding with coated electrodes.

#### 3.1.2. Metal Transfer, Melting Rate of Electrode, and Voltage Drop in the Anodic and Cathodic Region (Ua+c)

The way in which molten metal droplets transfer from the tip of electrode to the melting pool affects the operational aspects of welding, such as bead morphology, the amount of spatter, and even the stability of the process.

**Figure 8:**
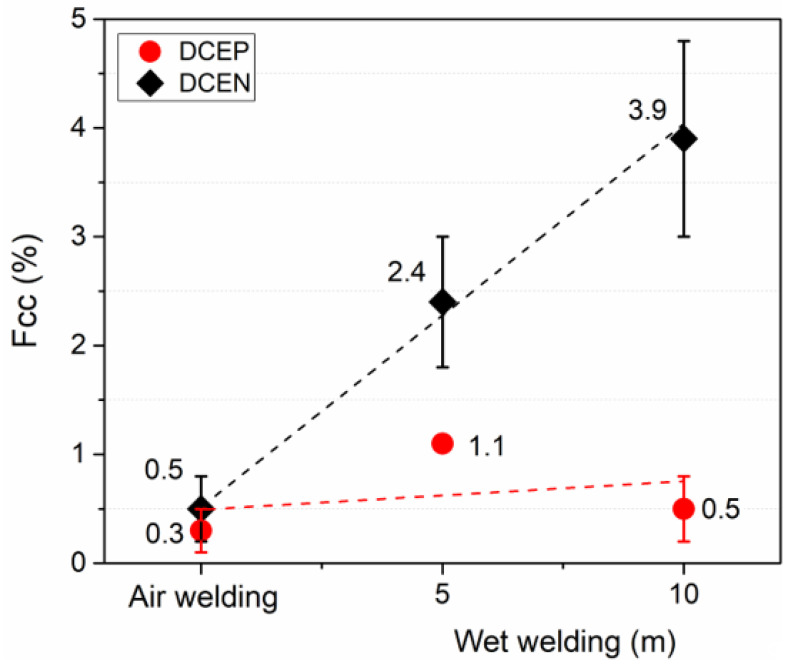
*Fcc* for rutile electrode in both polarities and for all conditions. Error bars show the standard deviation

In order to characterize the type of metallic transfer depending on the environmental condition and polarity, the short circuit factor (*Fcc*) was calculated. This index is based on the definition of the reference voltage, in order to define the separation between arc periods and short circuit periods. As shown in Figure 8, for the total sampling time (5 s) and using the rutile electrode in direct polarity (DCEN), the transfer mode was mainly by short circuit. Additionally, the tendency to increase short-circuits with depth (for DCEN) can be seen, thus reflecting the effect that hydrostatic pressure has on metal transfer. However, with the use of DCEP polarity, metal transfer occurred through a combination of globular/short circuit modes (mainly from the former), as reported previously by Uribe et al. (2017) [32].

**Figure 9:**
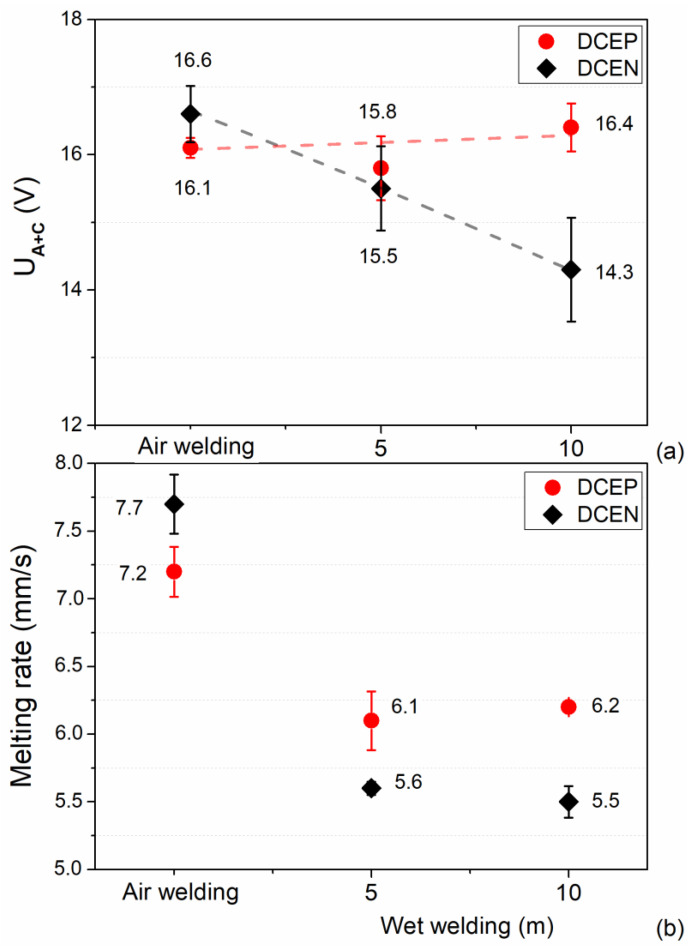
(**a**) Effect of polarity and hydrostatic pressure on the voltage drop in the anodic and cathodic connection (Ua+c). (**b**) Average melting rate for rutile electrode in both polarities and all conditions. Error bars show the standard deviation.

So far, our experimental results indicate that the underwater welding process with the rutile electrode is closely related to changes in the electric arc. Therefore, it is necessary—according to Scotti et al. (2006) [39]—to determine the particularities of the energy structure of the welding arc, as the energy consumed by a welding arc (for a given current value) is related to the total arc voltage. Indeed, Scotti et al. (2012) [21] mentioned that the most significant voltage drops in an arc are due to physical reactions that occur in arc–electrode and arc–workpiece connections. To determine the physical properties of the arc in UWW conditions, Figure 9a shows the voltage drop in the anodic and cathodic region (Ua+c) in shielded metal arc underwater welding arcs with short-circuiting transfer. In air welding, the electrode configuration at the DCEN condition resulted in a higher Ua+c value, compared to DCEP (approximately 0.5 V). Furthermore, in DCEP, the sum of the anode and cathode drops of the electric arc did not seem, in general terms, to be influenced by the depth. For DCEN, a reduction in the value of Ua+c of ~13% (2.3 V) was observed between air conditions and the depth of 10 m. Suga and Hasui (1990) [5] also analyzed the arc voltage for coated electrodes in DCEN, reporting that the sum of anode voltage drop and cathode voltage drop tended to decrease with increasing depth. Thus, this decrease in potential difference in the cathode and anode can serve as an essential factor in the drop in the welding voltage with the increase of depth.

Allum (1982) [19] mentioned that, in direct polarity, the arc is constricted and the cathodic region is concentrated at the tip of the electrode with increased pressure, resulting—according to Suga and Hasui (1990) [5]—in a decrease in melting efficiency. This behavior causes a drop in the welding voltage value associated with changes in the appearance of the arc and fluctuations in the welding voltage, as described by Allum (1982) [19]. Recently, Liberato et al. (2018) [38], from welding experiments with commercial coated electrodes up to a depth of 50 m, found that the welding voltage and the melting rate decreased with depth in DCEN polarity, obtaining lower values compared to those found in air welding.

As shown in Figure 9b, welds made in air welding conditions presented a behavior similar to that described in the literature, resulting in a higher melting rate in DCEN. It is evident that the electrode melting rate decreased with increasing depth, with the effect being more pronounced in DCEN. Richardson and Nixon (1985) [40] also reported a decrease in the melting rate of electrodes configured in the DCEN polarity in the hyperbaric MIG process (metal inert gas in dry conditions).

It appears that, for both works mentioned and in the results of this study, the melting rate of the electrode decreased with increasing depth, being the most striking effect for the rutile electrode in DCEN polarity. Furthermore, an increase in the number of short circuits with the pressure can be seen; therefore, with the DCEN polarity, an increase in pressure results in a drop in power generation in the cathode region and, so, the rate at which the consumable is melted decreases, generating a decrease in the welding voltage and naturally developing a higher number of short circuits.

With the use of DCEP, the metal transfer was characterized by the formation of larger drops of liquid metal which transfer to the melt pool at a low frequency. The previous statement is based on the evaluation of arc voltage oscillograms performed by Uribe et al. (2017) [32]. With DCEP polarity, the rutile electrode showed a tendency of increasing the arc voltage and the arc length with increasing pressure; Tsai and Masubuchi (1977) [14] mentioned that, with an increase in depth, the diameter of the arc column decreased, resulting in a higher arc voltage in order to guarantee the same amount of heat during the welding process.

### 3.2. Effect of the Pressure and Polarity on the Weld Bead Morphology of UWW

**Figure 10:**
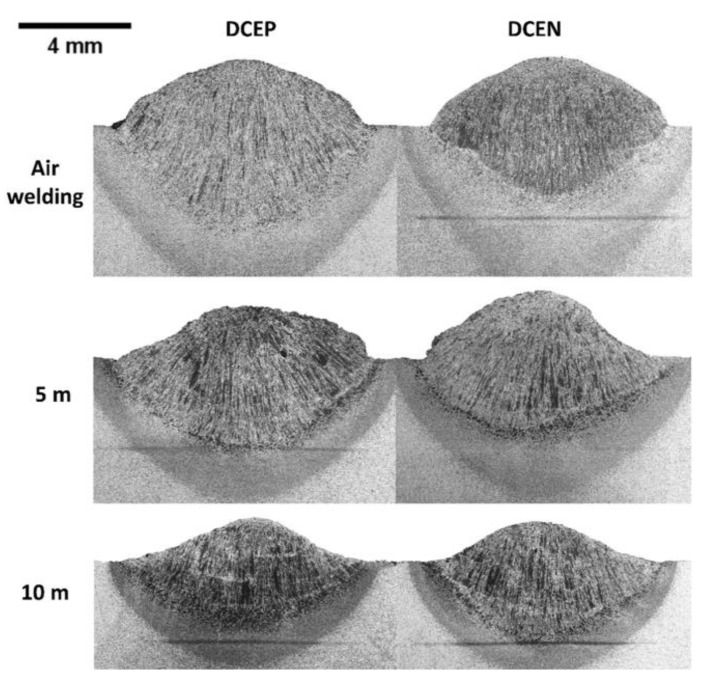
Macrographs of welds made with the rutile electrode. The black line is central segregation representing higher carbon content, which was diffused through an increase in temperature in the HAZ.

Figure 10 shows macrographs of the samples under study, indicating their relationships to the polarities and environmental conditions used. At this point, it is interesting to note that, for welds made in air welding conditions, the DCEP polarity setting provided the largest weld beads (i.e., the largest cross-sectional dimensions, compared to the electrode polarity configuration at the negative pole).

**Figure 11:**
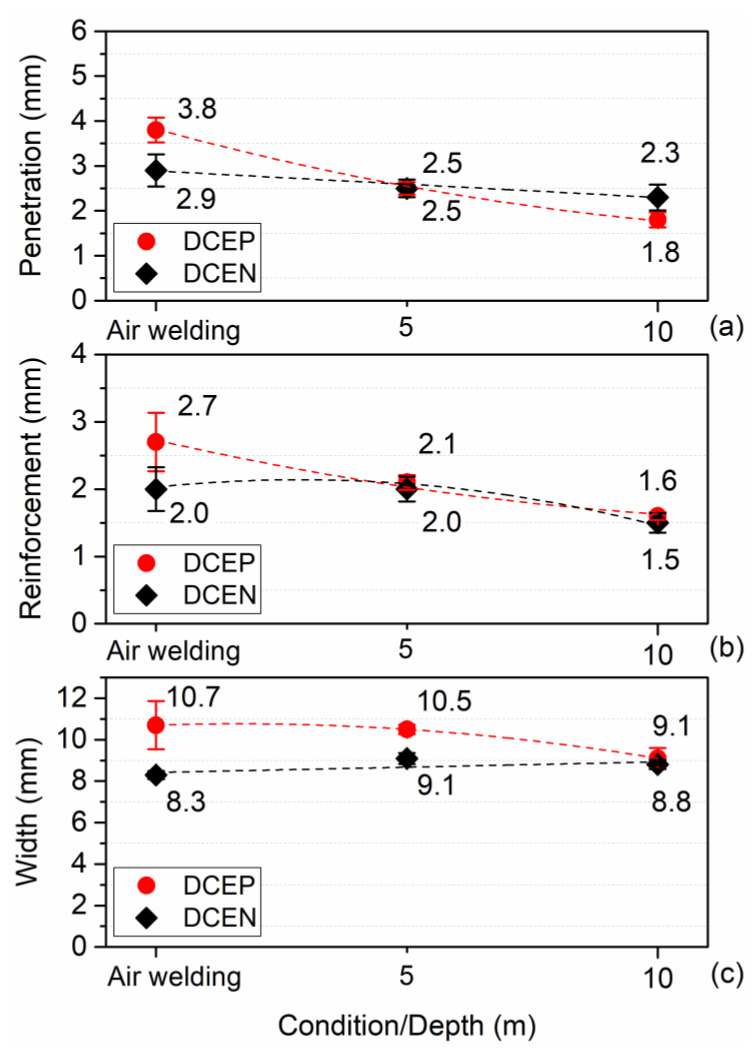
Averages values of weld bead penetration (**a**), reinforcement (**b**), and width (**c**) for rutile electrode in both polarities and for all conditions. Error bars show the standard deviation.

Figure 11 shows the average values of penetration (a), reinforcement (b), and width (c). For the DCEP polarity setting, it is possible to observe that the weld bead penetration decreased significantly (~35% on average) from air welding to 5 m water depth. On the other hand, from 5 to 10 m, the reduction in penetration reached a value of 30%; that is, the increase in depth caused a decrease in penetration. For DCEN polarity, there was a slight tendency to reduce penetration (12% and 8% from air welding to 5 m condition and from 5 to 10 m water depth, respectively). Taking into account that these values were within the corresponding standard deviations, it is not possible to conclude that there was a significant reduction in penetration with increasing depth under DCEN polarity. Thus, in general terms, penetration in DCEN polarity remained unchanged and only the values for DCEP polarity moved to a lower level. Moreover, when welding with DCEP polarity, the bead width was higher for up to 5 m in depth, compared to that with DCEN. From this point, with the increase in hydrostatic pressure (from 5 to 10 m), the width decreased, where it reached a value similar to that found in the weld bead obtained with the DCEN polarity. The width resulting from the DCEN polarity configuration remained broadly unchanged with increasing hydrostatic pressure.

Finally, it was observed that the reinforcement was greater in welds made in air welding conditions with DCEP. Welds made in DCEN polarity, on the other hand, did not show significant variation in reinforcement from air welding to wet conditions (5 m). At 10 m depth, the reinforcement decreased with increasing hydrostatic pressure and without the influence of polarity, as reported by Mazzaferro (1998) [13].

In DCEP polarity, the process was more sensitive to the effect of depth, as can be seen in the variation in weld bead geometry. For the 10 m depth, the reinforcement and width did not change with polarity. In relation to the penetration and the melting rate for the greatest depth used in the study (10 m), the penetration was higher in welds made with DCEN and the melting rate was lower in this polarity, according to the model by Tsai and Masubuchi (1977) [14]. It was also established that, for welds produced in the air welding condition (i.e., the starting point of comparison), it is possible to show how, in DCEP polarity, the increase in depth made the cross section of the weld bead smaller (i.e., penetration, width, and reinforcement were smaller from air welding to 5 m and decreased even more from 5 to 10 m). In turn, for welds made in DCEN, in general terms and taking into account statistical errors, only the reinforcement decreased to the simulated depth of 10 m.

Allum (1982) [19] had already observed that the behavior of the weld pool (in dry conditions) is influenced by pressure in several ways and, in turn, predicted that depth would have a strong effect on the geometry of the weld bead. In this sense, for the DCEN configuration, the drop of molten metal at the tip of the consumable periodically reaches the weld pool, causing short circuits and arc extinctions. The previous behavior generates a shorter established arc time (open arc) in relation to that of the DCEP polarity. Therefore, unlike DCEN, where the melting pool is disturbed continuously, there is mostly a globular transfer in DCEP. In this perspective, the pressure has a more significant effect on the plasma column and the resulting weld bead. As can be seen from Figure 11, the penetration, reinforcement, and width values decreased noticeably as a result of the use of DCEP and with the influence of increasing depth. With DCEN, there was a slight tendency to reduce the weld bead geometry (penetration, reinforcement, and width) with increasing depth; Menezes et al. (2019) [41] stated the electrodes that have a higher frequency of short circuits are more efficient, possibly due to lower losses of heat and material through spatters.

### 3.3. Closure

**Figure 12:**
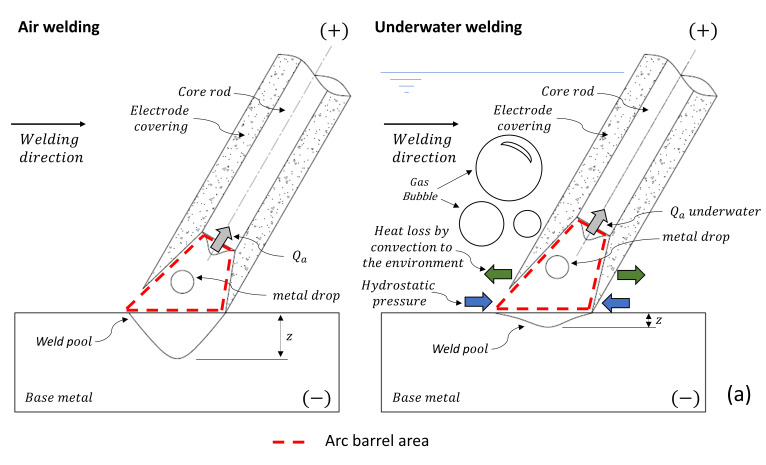

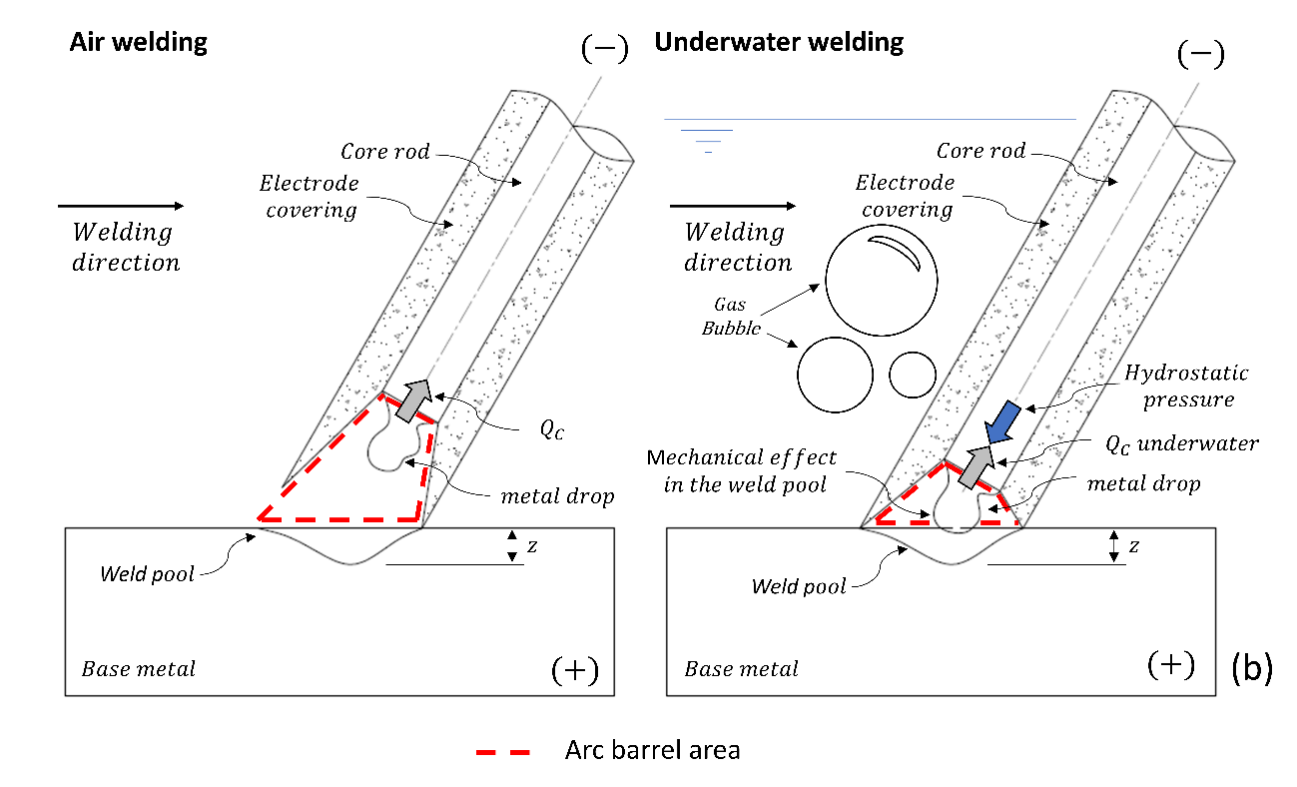
Schematic illustration of the effect of pressure on the arc phenomenon of UWW configured in reverse polarity (DCEP) (**a**) and direct polarity (DCEN) (**b**). *Qa* = heat generated in the anodic zone, *Qc* = heat generated in the cathodic zone, *z* = weld penetration.

Figure 12 shows the proposed models illustrating the electric arc phenomena and the resulting penetration of underwater wet welds depending on the polarity configuration.

Mazzaferro (1998) [13] mentioned that the hydrostatic pressure causes the arch column to constrict, resulting in a high current density and a high radial pressure. In the same line, Brown et al. (1974) [31] described that as the current density increases, the temperature increases, generating expansion in the arc column. Furthermore, the required operating voltage is also influenced by the heat exchange capacity of the medium that forms the arc, according to Scotti et al. (2012) [21], as higher losses result in higher voltages required to keep the arc in the same current. The previous descriptions support the diagram presented in Figure 12a and the results of this work: with increased hydrostatic pressure (blue arrows) and constriction of the plasma column, larger arc barrel areas (or arc length) are displayed, increasing the average welding voltage. Thus, with increasing depth, higher voltages are required to guarantee the current value imposed for the welding process. In this sense, higher welding voltages have less probability of metal transfer of the short-circuit type. According to Scotti et al. (2012) [21], when the arc length is increased, its surface area increases and, consequently, the heat losses increase; thus, an increase in arc length implies an increase in heat loss by convection in the plasma column (green arrows). Consequently, this reduces the heat transfer to the workpiece, resulting in the lower penetrations observed in underwater conditions and with the DCEP polarity setting.

For Brown et al. (1974) [31], one effect of depth is the constriction of the cathode spot, which generates—according to Suga and Hasui (1990) [5]—a decrease in fusion efficiency at the tip of the electrode. Consequently, in Figure 12b, it is possible to see schematically how the hydrostatic pressure (blue arrow) has an effect on heat generation in the cathodic region (gray arrow), resulting in a decrease in the energy originated in the arc–electrode connection (*Qc*). This results in a reduction in the electrode fusion rate, average welding voltage, and arc length. Thus, it is likely that two phenomena have an effect on the penetration of the weld bead with the use of the negative electrode: a thermal effect and a mechanical effect. In relation to the thermal effect, a shorter arc length minimizes radial heat losses in the plasma column, and thus more heat is transferred to the workpiece surface. Furthermore, in this study, it was evident that a lower rate of electrode consumption and a shorter arc length generated an increase in the number of short-circuit events. Thus, in view of the greater frequency of contact between the drop of metal on the tip of the consumable and the melting pool, a constant disturbance was established in the liquid weld metal, and it can be assumed that this behavior caused the weld bead morphology to be approximately invariable, compared to air welding conditions. This mechanical effect is similar to the forced-short circuiting transfer mode, as classified by Scotti et al. (2012) [42], where the force of the drops that strike the melt pool increases the penetration of the weld beads, notwithstanding this transfer mode occurring for high current levels.

## 4. Conclusions

The main results obtained in this paper are summarized as follows.

1. For the reverse polarity configuration (DCEP), an increase in hydrostatic pressure generates an increase in the operating voltage and in the arc length, consequently leading to radial heat losses in the plasma column as a result of the greater surface area, as compared to that under air conditions. This behavior generates a decrease in the cross section of the weld bead, as reflected in lower reinforcement, width, and penetration values.
2. In contrast, for the configuration of the electrode on the negative pole (DCEN), the hydrostatic pressure affects the generation of energy in the arc–electrode connection. This decreases the fusion efficiency, and therefore causes a drop in the average welding voltage with an increase in depth, together with a shorter arc length and an evident increase in the number of short-circuit events.
3. For DCEN, a shorter arc length minimizes radial heat losses in the plasma column, and thus more heat is transferred to the surface of the workpiece, developing a thermal effect on the weld bead geometry. At the same time, the occurrence of short circuits develops a mechanical effect on the melt pool, possibly guaranteeing that the weld bead geometry remains unchanged as the operating depth increases.
